# Betaine promotes cell differentiation of human osteoblasts in primary culture

**DOI:** 10.1186/s12967-017-1233-5

**Published:** 2017-06-07

**Authors:** Isabella Villa, Pamela Senesi, Anna Montesano, Anita Ferraretto, Fernanda Vacante, Alice Spinello, Michela Bottani, Simona Bolamperti, Alessandro Rubinacci, Livio Luzi, Ileana Terruzzi

**Affiliations:** 1 Bone Metabolism Unit, San Raffaele Scientific Institute, Milan, Italy; 2 Metabolism Research Center, IRCCS Policlinico San Donato, San Donato Milanese, Milan, Italy; 3 Department of Biomedical Sciences for Health, University of Milan, Milan, Italy; 4 Diabetes Research Institute, Metabolism, Nutrigenomics and Cellular Differentiation Unit, San Raffaele Scientific Institute, 60 Olgettina street, 20132 Milan, Italy

**Keywords:** Nutraceutical, Aging, IGF-I, Calcium signaling, Bone

## Abstract

**Background:**

Betaine (BET), a component of many foods, is an essential osmolyte and a source of methyl groups; it also shows an antioxidant activity. Moreover, BET stimulates muscle differentiation via insulin like growth factor I (IGF-I). The processes of myogenesis and osteogenesis involve common mechanisms with skeletal muscle cells and osteoblasts sharing the same precursor. Therefore, we have hypothesized that BET might be effective on osteoblast cell differentiation.

**Methods:**

The effect of BET was tested in human osteoblasts (hObs) derived from trabecular bone samples obtained from waste material of orthopedic surgery. Cells were treated with 10 mM BET at 5, 15, 60 min and 3, 6 and 24 h. The possible effects of BET on hObs differentiation were evaluated by real time PCR, western blot and immunofluorescence analysis. Calcium imaging was used to monitor intracellular calcium changes.

**Results:**

Real time PCR results showed that BET stimulated significantly the expression of RUNX2, osterix, bone sialoprotein and osteopontin. Western blot and immunofluorescence confirmed BET stimulation of osteopontin protein synthesis. BET stimulated ERK signaling, key pathway involved in osteoblastogenesis and calcium signaling. BET induced a rise of intracellular calcium by means of the calcium ions influx from the extracellular milieu through the L-type calcium channels and CaMKII signaling activation. A significant rise in IGF-I mRNA at 3 and 6 h and a significant increase of IGF-I protein at 6 and 24 h after BET stimulus was detected. Furthermore, BET was able to increase significantly both SOD2 gene expression and protein content.

**Conclusions:**

Our study showed that three signaling pathways, i.e. cytosolic calcium influx, ERK activation and IGF-I production, are enhanced by BET in human osteoblasts. These pathways could have synergistic effects on osteogenic gene expression and protein synthesis, thus potentially leading to enhanced bone formation. Taken together, these results suggest that BET could be a promising nutraceutical therapeutic agent in the strategy to counteract the concomitant and interacting impact of sarcopenia and osteoporosis, i.e. the major determinants of senile frailty and related mortality.

## Background

Betaine (BET) is an important human nutrient [1], originally identified during the extraction of sucrose from sugar beets (*Beta vulgaris*). It was then found that BET is widely distributed in many marine invertebrates, plants, and animals. BET physiologically functions as either an organic osmolyte to protect cells under stress or as a methyl donor via trans-methylation [2]. Extensive in vitro and in vivo studies suggested that dietary BET supplementation was effective in reducing body fat deposition, improving hepatic steatosis [3], and in increasing lean mass gains [4]. Several investigations reported in fact significant effects of BET on sports performance and body composition [3–5]. However, the mechanisms underlying these effects have not been defined yet.

As recently reviewed [4], BET favours lipolysis and inhibits lipogenesis via gene and protein expression; it promotes the autocrine/endocrine release of insulin-like growth factor type I (IGF-I), and modulates its relative receptor signaling pathway. In particular, it has been shown that BET enhances neo myotubes formation and differentiation while promoting IGF-I gene and protein expressions in C2C12 murine myoblasts [6], a model of skeletal muscle development [7]. IGF-I has long been known to play a role not only in muscle hypertrophy but even in bone strength [8, 9]. In bone, IGF-I stimulates osteoblast differentiation and bone formation through the activation of the mammalian target of rapamycin (mTOR) pathway [10]. Indeed, IGF-I stimulates RUNX2 downstream gene expression, a critical step in osteogenesis, by up-regulating the protein levels of PI3K subunits, Akt, and increasing p70S6 kinase (p70 S6K) thus stimulating osteoblast differentiation, protein synthesis and cell growth [11]. The critical role of IGF-I in bone is further supported by the inhibition of osteoblast differentiation, protein synthesis and cell growth after treating the cells with antibodies against IGF-I [9–11].

Skeletal muscle and bone are of mesodermal origin and osteogenesis and myogenesis process are characterized by a close interaction and several common molecular mechanisms [12–14]. Moreover, numerous factors enhance both myogenic and osteogenic differentiation, including IGF-I and calcium. IGF-I has long been known to play a role in muscle hypertrophy and bone strength. Based on this evidence and on our encouraging results obtained treating myostasts with BET, we have hypothesized that BET might stimulate the differentiation of bone cells [6]. Osteoblast differentiation [15] is a multi-step process, where mesenchymal cells differentiate along the osteoblast lineage until the osteocyte state. RUNX2 is the main transcription factors inducing the osteogenic process [16] by activating other important osteoblast-specific transcription factors, like osterix (OSX) [17, 18]. During the differentiation of pre-osteoblasts into mature osteoblasts, RUNX2-OSX axis regulates the expression of bone marker proteins, including type I collagen, bone sialoprotein (BSP) and osteopontin (OPN) [15, 16, 19]. The effects of RUNX2 in bone development is positively regulated by ERKs cascade. In fact, differentiation of human marrow stromal cells to osteoblasts is associated to ERK1/2 phosphorylation while the inhibition of both ERK1 and ERK2 activities by the dominant negative ERK1 protein prevents differentiation of human osteoblasts [20] and shifts human marrow stromal cells to adipogenesis [21].

Based upon these considerations, we have investigated if BET could induce osteoblast differentiation by evaluating the expression of osteogenic genes, i.e. RUNX2, OSX, BSP and OPN, and if it could activate important intracellular pathway such as ERK pathway, protein synthesis and calcium signaling, which are fundamental for the recruitment and function of bone cells [22]. In this preliminary evaluation of the pathways involved in the potential osteogenic effect of BET, we extended our investigation on the potential anti-oxidative effect of BET [23]. Since Superoxide Dismutase 2 (SOD2), a crucial mitochondrial enzyme playing a role in antioxidant activity [24], is associated to intracellular calcium perturbations [25], we have evaluated SOD2 gene expression in human osteoblasts after BET treatment.

The present study could provide new insights on the potential function of BET as a nutraceutical supplementation in the strategy to counteract the concomitant and interacting impact of sarcopenia and osteoporosis, i.e. the major determinants of senile frailty and related mortality [26].

## Methods

### Materials

Fura-2 acetoxymethyl ester (Fura-2/AM) was from Calbiochem (La Jolla, CA, USA). Bis-(1,3-dibutylbarbituric acid) trimethine oxonol, DiBAC_4_-(3), was obtained from molecular probes (Eugene, OR, USA). All reagents, including materials for video imaging experiments, were purchased from Sigma Chemical Co. (St. Louis-MO, USA).

Primary antibodies against: Calnexin (H-70), GAPDH (FL-335), CaMKII (M-176), pCaMKII (Thr286), p70 S6 (C-18), pp70 S6 (C-18), ERK1 (K-23), ERK2 (C-14), pERK1/2 (E-4), OPN (K-20), peroxidase-conjugated secondary antibodies for western blot analysis and FITC or Rhodamine-conjugated antibodies for immunofluorescence study were purchased from Santa Cruz Biotechnology (Heidelberg, Germany). AlexaFluor^®^488-Phalloidin was obtained from Life Technologies (Carlsbad-CA, USA).

### Human osteoblast-like cells (hOBs) cultures

Human bone cell cultures were established by means of a modified version of the Gehron-Robey and Termine procedure [27] using trabecular bone samples obtained from waste material of female patients during orthopedic surgery for degenerative diseases or traumatic fractures of the femoral neck requiring osteotomy. None of the patients (aged 71–82 year) had any malignant bone diseases and all of them gave their written consent for the use of the waste material. The protocol was approved by the Institutional Ethical Committee (Protocol BMU-WNT, 25.03.2008; amendment 1, 29.9.2012). No significant trend related to donor age was observed in any of the effects studied. Briefly, the trabecular bone was cut into small pieces and thoroughly washed with commercial standardized Joklik’s modified MEM serum-free medium, to remove no adherent marrow cells. The pieces were incubated with rotation at 37 °C for 30 min with the same medium containing 0.5 mg/ml type IV collagenase, and collagenase digestion was stopped by the addition of Iscove’s modified medium containing 10% fetal bovine serum (FBS). Between eight and ten pieces from each patient were then placed in 25 cm^2^ flasks and cultured in IMDM containing 10% FBS, 100 U/ml penicillin, 100 µg/ml streptomycin, 50 U/ml mycostatin, and 0.25 µg/ml amphotericin B until confluence; the culture medium was changed every 2–3 days. The cell population was tested for alkaline phosphatase (ALP) and osteocalcin (BGP) production after 1,25(OH)_2_D_3_ 10^−8^ M to ensure that the cells were endowed with osteoblast characteristics. ALP and BGP were measured by means of a multianalyzer COBAS (Roche Diagnostics SpA, Monza, Italy). Cells were used at first passage to reduce the possibility of phenotype changes.

### Reverse transcription and semi-quantitative real time PCR

After 24 h of serum starvation, confluent hObs were treated with BET (10 mM) for 1, 3, 6 and 24 h. The relative expression of osteogenic genes RUNX2, OSX, BSP, OPN was evaluated. IGF-I and SOD2 mRNA levels were also evaluated. Total RNA from confluent hOBs was extracted using TRIzol reagent (Thermo Fisher Scientific Inc., Waltham, MA USA), according to the manufacturer’s instructions. 1 µg of total RNA was reverse transcribed to cDNA using oligodT primers and M-MLV reverse transcriptase (Promega Corporation, Madison, WI, USA). 10 µg of cDNA were subjected to real-time PCR reactions using primer-probe sets validated and purchased as “Assay-on-Demand” from Applied Biosystems (Thermo Fisher Scientific Inc., Waltham, MA USA) in singleplex PCR mix. Real time PCR reaction was performed in an ABI PRISM^®^ 7900 Sequence Detection System (Thermo Fisher Scientific Inc., Waltham, MA USA). Each gene expression was first normalized with β-actin content and the relative quantification treated/untreated was calculated with the 2^−ΔΔCt^ method. Three replicates were performed for each experimental point and experiments were repeated several times with cells obtained from different donors.

### Western blot analysis

After 24 h of serum starvation, confluent hObs were treated with BET (10 mM) for 5, 15 and 60 min or for longer time (6–24 h). At the end of the experiments, hOBs cells were homogenized in RIPA lysis buffer supplement with protease inhibitors as described [28]. Aliquots of 35 μg supernatant proteins, quantified using Bradford method, were resolved by SDS-PAGE. Electrophoresed proteins were transferred to nitrocellulose membrane (Protran^®^, Whatman^®^ Schleicher & Schuell, Sigma Chemical Co., St. Louis-MO, USA). The membranes were incubated with specific primary antibodies and then with HRP conjugated anti species-specific secondary antibodies. The protein signals were normalized using the relevant calnexin or anti GAPDH bands. Immunoreactive bands were visualized by an enhanced chemiluminescence method (Amersham Pharmacia Biotech, Piscataway, NJ, USA). Bands on X-ray films were then quantified using Scion Image software (Scion Corp., Frederick, MD, USA). The data were then converted to fold change (FC) of the control.

### Immunofluorescence analysis

After 24 h of serum starvation, hObs were treated for 3, 6 and 24 h with BET (10 mM). hObs cells, fixed and permeabilized as previously described [28], were blocked with PBS containing 1% bovine serum albumin. Cells were then immunostained with specific antibodies FITC or rhodamine conjugated and nuclei-revealed with DAPI staining. Slides were mounted with Moviol. Cells were observed using Nikon Eclipse 50I microscopy and images were captured using Nis-Elements D 4.00 software (Nikon Instruments Europe BV, Netherlands). Data were displayed and analyzed using Adobe Photoshop CS4. Automated quantification on immunofluorescence signal was performed by using Image J program (http://imagej.nih.gov/ij/) [29].

### Intracellular calcium determination at a single cell level

BET possible action on the intracellular calcium concentration ([Ca^2+^]_i_) was studied in two different experimental condition: a physiological extracellular calcium concentration ([Ca^2+^]_o_ = 2 mM) and a nominally extracellular calcium free condition. In the first condition all the experiments have been performed in Krebs–Ringer-HEPES solution (KRH, NaCl 125 mM, KCl 5 mM, KH_2_PO_4_ 1.2 mM, MgSO_4_ 1.2 mM, CaCl_2_ 2 mM, glucose 6 mM and HEPES 25 mM, pH 7.4). Nominally calcium free condition consisted in a KRH lacking of CaCl_2_, without calcium chelators to avoid cytotoxic effects [30]. Possible calcium ions residues derived by the doubly distilled water. After 24 h serum starvation, cells, seeded on a glass coverslip (24 mm diameter, thickness 0.13–0.17 mm, VWR International, West Chester, Pennsylvania, USA), were incubated for 20 min at 37 °C with 2.5 µM Fura-2/AM acetoxymethyl ester (Fura-2/AM) and 2.5 µM Pluronic F-127 in 1 ml KRH. At the end of Fura-2/AM loading, the glass coverslips were placed on a thermostated (TC-202 A) PDMI-2 perfusion chamber (Medical System Corporation, Harvard Apparatus, Holliston, MA, USA), positioned on a microscope stage (TE 200, Nikon, Tokyo, Japan) connected to a CCD intensified camera (Extended Isis, Photonic Science, Millham, UK). The intracellular calcium concentration in single cells was continuously monitored using the fluorescence image acquisition and data analysis system supplied by applied imaging (High Speed Dynamic Video Imaging Systems, Quanticell 700, Sunderland, UK). The intracellular free calcium concentration, [Ca^2+^]_i_, was derived from a calibration performed with external standards of calcium and Fura-2/AM applied to the 340/380 nm images [31]. BET was administered to cells in a single dose of 10 mM. The single-cell analysis provided the following results: (i) the percentage of responsive cells, considering only cells which responded to BET administration with [Ca^2+^]_i_ increments equal or above 20 nM; (ii) the mean [Ca^2+^]_i_ rise, derived from the subtraction of the baseline value to the peak value after BET administration for each cell and then averaged for all the analyzed cells.

### Experiments with promoters and/or inhibitors of [Ca^2+^]_i_ rise

To study [Ca^2+^]_i_ changes in nominally calcium free conditions, 10 µM Thapsigargin from a DMSO stock solution was used before and after BET administration. Thapsigargin is a specific endoplasmic calcium pump inhibitor, which induces an immediate release of the stored intracellular calcium. In order to determine BET effect on calcium entry from the extracellular buffer, agonists and antagonists of cellular membrane calcium channels were used in physiological condition. Bay-K 8644, a known L-type Ca^2+^ channels (VDCCL) agonist, was solubilized in DMSO and administered to hOBs at 400 nM concentration. The dihydropyridine derivatives Nimodipine and Nifedipine, two known antagonists of VDCCL [32], were solubilized in DMSO and were administered to cells (30 µM) 10 min before BET administration. LaCl_3_, known to block the cell membrane Ca^2+^ channels in an irreversible manner and prevent any Ca^2+^ movement across the membrane [33, 34], was used at 250 µM concentration 5 min before BET administration. MnCl_2_ was used to evaluate calcium entry from the extracellular medium due to its ability of quenching the fluorescence emission at 360 nm, the wavelength at which the excitation of Fura-2/AM is independent from the presence of calcium [35].

### Measurement of membrane potential

The measurement of the membrane potential before and after stimulus was undertaken by using DiBAC_4_-(3), a lipophilic, negatively charged slow potential-sensitive oxonol dye [36], which responds to membrane depolarization moving from the extracellular medium into the cytosol. The increased intracellular concentration of the dye results in an increase of the fluorescence emission [37]. Cells were loaded with 500 nM DiBAC_4_-(3) in KRH solution and incubated for 15 min at 37 °C under 300 rpm shaking in a Thermomixer (Eppendorf s.r.l. Milan, Italy). At the end of incubation, fluorescence was recorded (λ_ex_ 490, λ_em_ 510) and defined as “Before stimulus”. Then, cells were treated with 10 mM BET, 1 µM Gramicidin or a Hyperpolarizing KRH solution (0 mM NaCl, 5 mM KCl). Fluorescence was immediately recorded in the case of BET and Hyperpolarizing KRH solution and after 5 min in the case of Gramicidin. In all these samples, fluorescence was defined as “After stimulus”.

### Statistical analysis

Data are presented as the mean ± SD of experiments performed three–nine. Statistical analysis were performed with specific statistical packages (Prism v 7.00 GraphPad Software, San Diego, CA, USA and SPSS 20 statistical software, Chicago, IL, USA). Statistically significant differences were determined using *t* student test, ANOVA tests (nonparametric ANOVA test-Kruskal–Wallis test) followed by appropriate multiple-comparison test: Dunn’s post test, differences were considered significant when p ≤ 0.05.

## Results

### Betaine effects on osteogenic gene and protein expression in human osteoblasts

**Fig. 1:**
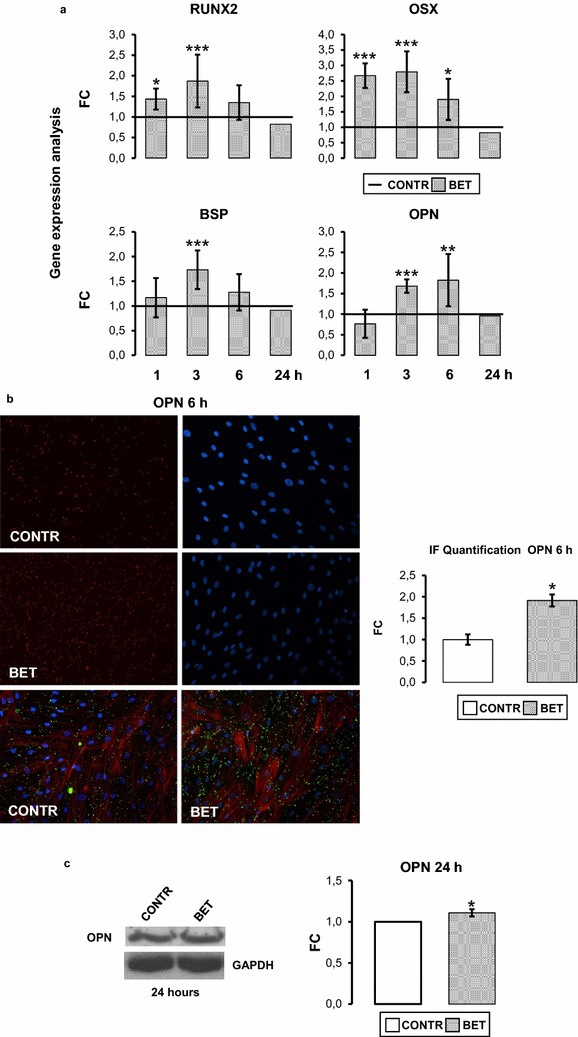
Effects of Betaine on osteogenic gene and protein expression in human osteoblasts. **a** Real time PCR assay: 10 mM BET stimulated significantly, compared to control (CONTR), the gene expression of RUNX2 at 1 and 3 h, of OSX at 1, 3 and 6 h, of BSP at 3 h and of OPN at 3 and 6 h. **b** Immunofluorescence staining of OPN protein after 6 h of 10 mM BET treatment and relevant quantification showing an increase in OPN protein compared to control (CONTR). Osteoblastic morphology was analyzed by Phalloidin (*red*) and OPN (*green*) staining (magnification: 20×). **c** Representative western blot and relevant quantification of OPN protein content increased after 24 h of 10 mM BET treatment. Data are the mean ± SD of six to nine experiments performed with cells obtained from different donors. ANOVA for non parametric data (Kruskal–Wallis) with Dunn’s multiple comparison test: *p ≤ 0.05, **p ≤ 0.01, ***p ≤ 0.001 vs control (CONTR). For western blot-immunofluorescence studies, Student’s *t* test: *p ≤ 0.05

Cell culture responded to 24 h 1,25(OH)_2_D_3_ (10^−8^ M) treatment with a significant increase of ALP and BGP, assuring that the cultures were endowed with osteoblastic characteristics. Basal and stimulated ALP activity was respectively 47.20 ± 10.68 and 69.91 ± 12.72 UI/mg protein (p < 0.001), and BGP values were 8.43 ± 0.98 and 27.63 ± 2.99 ng/mg protein (p < 0.001). To investigate the role of BET on osteoblast differentiation, hOBs were cultured with 10 mM BET for 1, 3, 6 and 24 h. We firstly investigated BET action on crucial osteoblastic transcription factors: RUNX2 and OSX [15–19]. The real time PCR analysis showed that RUNX2 and OSX mRNAs expression was significantly increased after 1 h (p < 0.05; p < 0.001) and 3 h (p < 0.001; p < 0.001; Fig. 1a), OSX gene expression was significantly enhanced not only at 1 and 3 h but also after 6 h (p < 0.05, Fig. 1a). Because the expression of many osteogenic proteins, including OPN and BSP, is directly controlled by RUNX2-OSX axis [18, 19], we determined BSP and OPN expression levels: BSP and OPN mRNA expressions increased significantly after 3 h of BET treatment (p < 0.001) and the increase of OPN mRNA lasted until 6 h (p < 0.01 Fig. 1a). After 24 h, all gene expression levels returned to control level (Fig. 1a). We also evaluated OPN protein levels. Immunofluorescence (IF) and western blot analyses revealed an increase of OPN protein level after 6 and 24 h (p < 0.05) of treatment (Fig. 1b, c).

### Intracellular pathways activated by Betaine: Betaine stimulates ERKs and IGF-I pathways

**Fig. 2:**
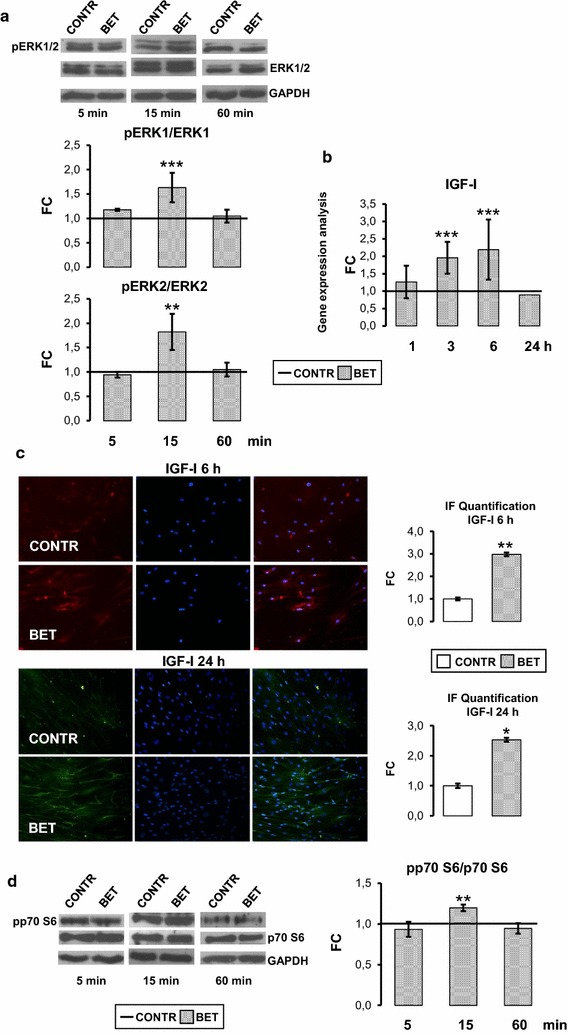
Betaine activates ERKs pathway and IGF-I gene and protein expression. **a** Representative western blot and relevant quantification of ERK pathway activation: in hObs treated with 10 mM BET, ERK1/2 activation peak was observed at 15 min. **b** 10 mM BET increased significantly IGF-I gene expression respect to control (CONTR) at 3 and 6 h, as measured by real time PCR. **c** Immunofluorescence study and relevant quantification showed an increase in the number of IGF-I positive cells in BET condition at 6 and 24 h (magnification: 20×). **d** Representative western blot and relevant quantification: after 15 min, BET induced a significant activation of p70 S6 kinase. Data are the mean ± SD of six experiments performed with cells obtained from different donors. ANOVA for non parametric data (Kruskal–Wallis) with Dunn’s multiple comparison test: ***p ≤ 0.001 vs control. For western blot-immunofluorescence studies, Student’s *t* test: *p ≤ 0.05, **p ≤ 0.01, ***p ≤ 0.001

To further elucidate the osteogenic effect of BET, we evaluated ERKs activation. Western blot results showed that BET (10 mM) increased significantly the phosphorylation of ERK1 (p < 0.001) and ERK2 (p < 0.001) after 15 min as shown by the ratio between their phosphorylated and not phosphorylated forms (Fig. 2a). BET (10 mM) also significantly stimulated one of the major activator of ERK, IGF-I. The gene expression of IGF-I increased significantly after 3 and 6 h (p < 0.001; Fig. 2b) and returned to control level after 24 h, whereas IGF-I receptor mRNA levels were not affected by BET treatment (data not shown). Accordingly, immunofluorescence for IGF-I showed an increase in IGF-I-positive cells after 6 h (p < 0.001) and 24 h (p < 0.05; Fig. 2c). BET was also able to stimulate the activation levels of p70 S6 kinase after 15 min (p < 0.001; Fig. 2d).

### Intracellular pathways activated by Betaine: Betaine induces calcium influx

**Fig. 3:**
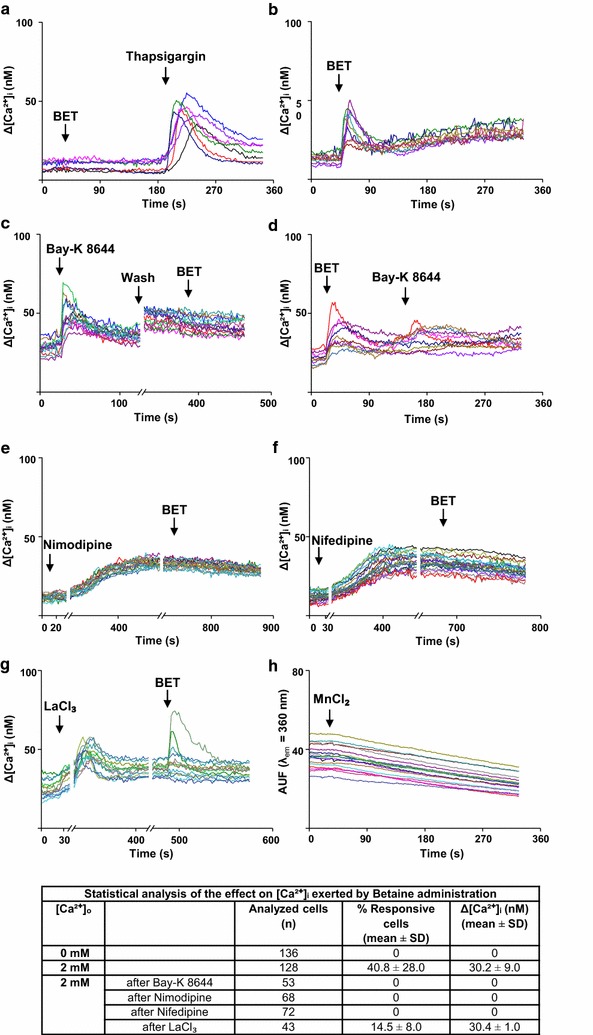
Betaine induces calcium influx from the extracellular milieu. **a** 10 mM Betaine in nominally free [Ca^2+^]_o_, followed by 10 µM Thapsigargin. **b** 10 mM Betaine in 2 mM [Ca^2+^]_o_. **c** 400 nM Bay-K 8644, wash and restoration of the initial condition (time break axis), followed by 10 mM Betaine in 2 mM [Ca^2+^]_o_. **d** 10 mM Betaine followed by 400 nM Bay-K 8644 in 2 mM [Ca^2+^]_o_. **e** 10 min incubation (time break axis) with 30 µM Nimodipine, followed by 10 mM BET administration (2 mM [Ca^2+^]_o_). **f** 30 µM Nifedipine incubation for 10 min (time break axis) and subsequent treatment with 10 mM BET (2 mM [Ca^2+^]_o_). **g** Administration of 250 µM LaCl_3_ and 5 min incubation (time break axis), followed by 10 mM BET (2 mM [Ca^2+^]_o_). **h** Fluorescence intensity changes of Fura-2/AM recorded at the calcium insensitive excitation wavelength of 360 nm (λ_em_ = 510 nm), after the administration of 2 mM MnCl_2_ in presence of 10 mM BET (2 mM [Ca^2+^]_o_). These results lead to the hypothesis that BET induced calcium influx through L-type voltage-dependent Ca^2+^ channels. All the stimuli were added at the time points indicated by the *arrows in the graphs*. Each graph shows the intracellular calcium changes of a cells representative group from at least 3–6 independent experiments. *Each line* in the graphs display a single cell behavior. The table reported in the graph summarizes the number of analyzed cells together with the percentage of responsiveness and the mean [Ca^2+^]_i_ increases for all the experiments here described

Since ERKs signaling can be modulated by calcium signaling, a key parameter for the regulation of osteoblastic differentiation [22, 38, 39], we analyzed the effect of BET on calcium fluxes. The administration of 10 mM BET to hOBs in nominally calcium free condition did not induce any cytosolic calcium rise. The subsequent administration of Thapsigargin, a drug able to empty the calcium stores from the cytosolic endoplasmic reticulum, resulted in an increase of [Ca^2+^]_i_, thus demonstrating the full viability of the cells as well as the integrity of their calcium stores (Fig. 3a, Table). The administration of BET (10 mM) in presence of 2 mM [Ca^2+^]_o_, caused a [Ca^2+^]_i_ rise in 40.8 ± 28.0% of the analyzed cells (Fig. 3b, Table), suggesting a role of BET in inducing a calcium influx into hOBs from the extracellular milieu. The next step was to explore BET mechanism inducing calcium influx into hOBs. At first, the possible involvement of L-type calcium channels (VDCCL) was considered. These channels are known to be differently expressed during hOBs differentiation [40, 41] and their functionality is modulated by 1,25(OH)_2_ vitamin D3 [42]. When an agonist of VDCCL, Bay-K 8644 was added to hOBs, an increase of [Ca^2+^]_i_ was recorded indicating the presence of active VDCCL in hOBs. The same cells, stimulated with BET after a wash and restoration of initial experimental conditions, failed to respond with an increase in the [Ca^2+^]_i_ (Fig. 3c, Table). Similarly, when BET was first administered to hOBs, Bay-K 8644 did not produce any [Ca^2+^]_i_ increase (Fig. 3d, Table). These observations lead to the conclusion that BET induces calcium influx from the same VDCCL activated by Bay-K 8644. Another demonstration of VDCCL involvement came from the use of Nimodipine and Nifedipine, known inhibitors of these channels. Unexpectedly, the administration of both inhibitors in hOBs produced a slow and slight increase in the [Ca^2+^]_i_ with a plateau after 400 s. The possible [Ca^2+^]_i_ increase following these inhibitors was described before in SaOS-2 cells [43]. After the inhibition of VDCCL by Nimodipine and Nifedipine, BET addition did not exert any [Ca^2+^]_i_ change (Fig. 3e, Table). Since both agonist (Bay-K 8644) and antagonists (Nimodipine and Nifedipine) abolished BET effect on hOBs, it is conceivable that BET determines a calcium influx acting on the L-type calcium channels. To better understand the origin of BET-induced calcium influx, LaCl_3_, an inorganic inhibitor of calcium entry [34], was pre administered to BET treated hOBs, a reduction of [Ca^2+^]_i_ was observed (Fig. 3f, Table). The final confirmation of a calcium influx from the extracellular buffer induced by BET came from the quenching of Fura-2/AM fluorescence, using 2 mM MnCl_2_. Mn^2+^ ions are able to pass the cell membrane through the same channels involved in the Ca^2+^ entry. Once inside the cells, Mn^2+^ ions bind to Fura-2/AM and their presence is recorded by the fluorescence emission quenching due to the excitation at 360 nm, the wavelength at which the excitation of Fura-2/AM is independent from the presence of calcium [35]. When MnCl_2_ was added to hOBs 10 s after BET, a reduction of Fura-2/AM fluorescence due to an influx of Mn^2+^ ions was recorded (Fig. 3h).

### Betaine depolarizes hObs membrane

**Fig. 4:**
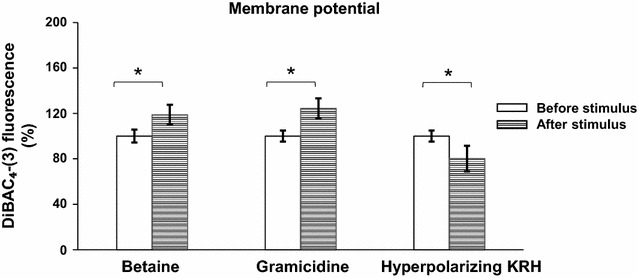
Betaine evokes hOBs membrane depolarization. DiBAC_4_-(3) (500 nM) in KRH solution (2 mM CaCl_2_) assay: BET induced membrane depolarization. The *graph* represents the fluorescence intensity variations observed in cells treated with 10 mM BET, 1 µM Gramicidin or Hyperpolarizing KRH solution. Each *bar* represents the mean value ± SD of three analogous experiments. Student’s *t* test: *p < 0.05 between fluorescence value recorded before and after the administration of one stimulus

Since VDCCL are defined as voltage-operated calcium channels, a possible direct effect of BET on membrane depolarization was considered. The cell membrane potential was measured by the use of DiBAC_4_-(3), a fluorescent probe whose fluorescence intensity increases in depolarized conditions while decreases in hyperpolarizing conditions. The hObs basal membrane potential was considered as “Before stimulus” and indicated as 100%. The addition of 10 mM BET increased by 18% the fluorescence intensity, indicating a depolarizing effect, as what recorded with Gramicidin (plus 23%), a known depolarizing agent, and opposite to what recorded (minus 19%) with KRH Hyperpolarizing buffer (Fig. 4).

### Betaine enhances CaMKII signaling pathway

**Fig. 5:**
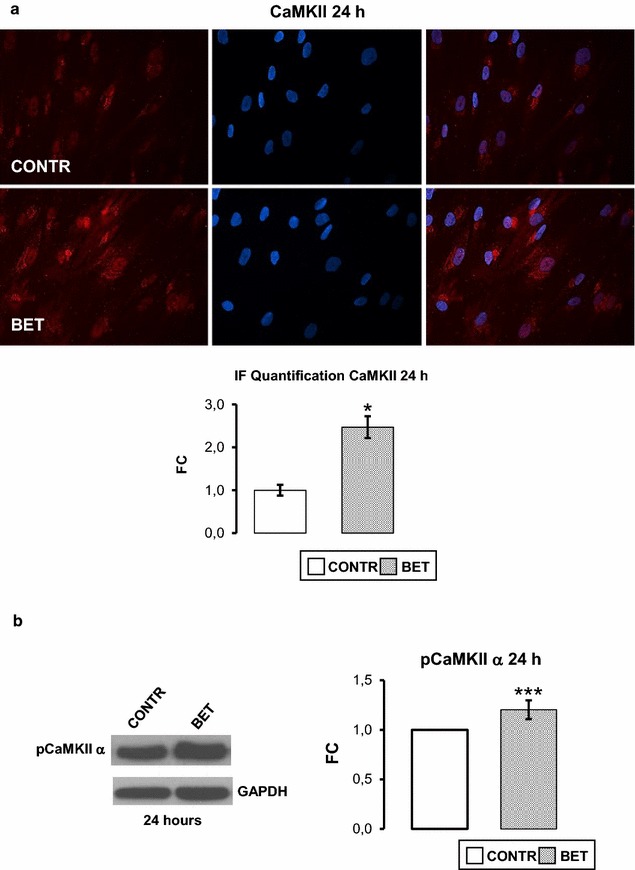
Betaine enhances CaMKII signaling pathway. **a** Immunofluorescence study (magnification: 40×) and relevant quantification: BET stimulated hObs showed a higher CaMKII positive cell number compared to control. **b** Representative western blot and relevant quantification: BET stimulus for 24 h promotes CaMKII α isoform activation. Data are the mean ± SD of six experiments performed with cells obtained from different donors. Student’s *t* test: *p ≤ 0.05, ***p ≤ 0.001

Since BET increased [Ca^2+^]_i_ through VDCCL channels, the possible effect of 10 mM BET on Ca^2+^/calmodulin-dependent kinase II (CaMKII) was evaluated. BET significantly increased CaMKII positive cells number (p < 0.05) after 24 h of stimuli, as shown by immunofluorescence analysis (Fig. 5a). Moreover, several works have demonstrated that CaMKII isoform plays a critical role in osteoblastic differentiation regulating the activation of many transcription factors including OSX [39, 44]. BET significantly (p < 0.001) stimulated the phosphorylation of CaMKII isoform as measured 24 h after treatment by western blot analysis (Fig. 5b).

### Betaine enhances SOD2 levels

**Fig. 6:**
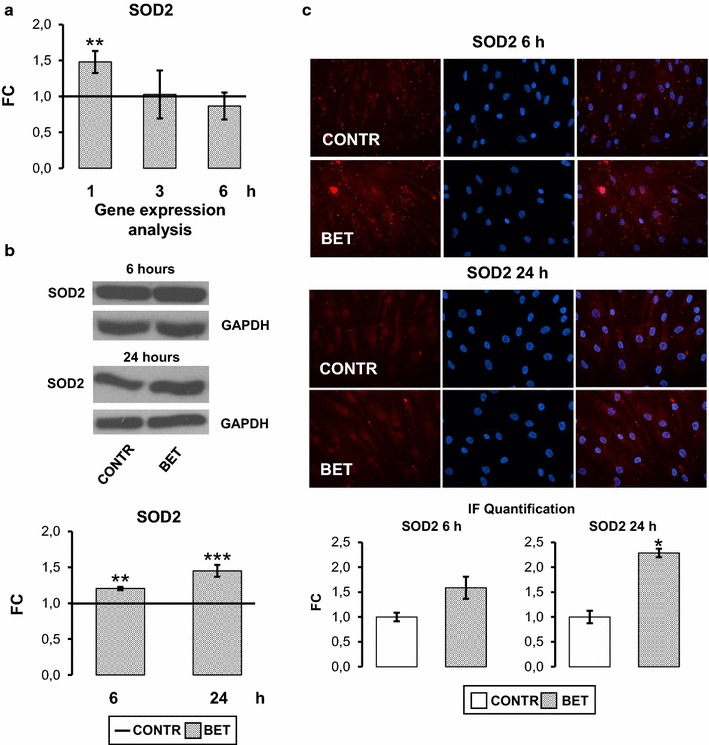
Betaine increases SOD2 levels. **a** Real time PCR. 10 mM BET up regulated significantly SOD2 gene expression after 1 h. **b** Western blot analysis and relevant quantification: BET increased significantly SOD2 protein content in hObs after 6 and 24 h treatment. **c** Immunofluorescence analysis (magnification: 40×) and relevant quantification confirmed BET positive action on SOD2 protein level. Data are the mean ± SD of six experiments performed with cells obtained from different donors. ANOVA for non parametric data (Kruskal–Wallis) with Dunn’s multiple comparison test: **p ≤ 0.01 vs control. For western blot-immunofluorescence studies: Student’s *t* test: *p ≤ 0.05, **p ≤ 0.01, ***p ≤ 0.001

Finally, we estimated BET ability to promote anti-oxidative stress cellular machine. As reported in Fig. 6a, BET significantly (p < 0.01) up regulated SOD2 mRNA expression after 1 h of treatment, that returned at basal values after 3 and 6 h. This effect was associated with a significant increase after 6 (p < 0.01) and 24 h (p < 0.001) also in SOD2 protein content (Fig. 6b, c) as shown by western blot and immunofluorescence assay.

## Discussion

In this study, we have shown that BET favours human osteoblast differentiation by promoting osteogenesis related gene expression such as RUNX2, OSX, OPN and BSP. Our analysis revealed that the osteogenic effect of BET involves the activation of calcium and ERK signaling, associated with an increased production of IGF-I. Calcium signaling occurred via L-type voltage-dependent Ca^2+^ channels and that it was associated with the depolarization of the cell membrane.

The present results are in line with our previous study showing that BET acts early in the process of myogenesis to determine cell fate [6]. We there used an in vitro murine model of myoblasts that, upon BET, exhibited an enhancement in the formation of neo myotubes, through the activation of IGF-I signaling. With the current study, we further evaluated BET role, by outlining its potential effect in human osteoblasts. Since BET up regulates RUNX2 and OSX gene expression and promotes the synthesis of specific non collagenous bone proteins, like BSP and OPN, it is likely that this nutrient is involved in triggering major events in osteogenesis and bone remodeling, potentially restoring the negatively affected balance of bone remodeling during aging. RUNX2 and OSX are indeed key transcription factors of osteoblastic differentiation which activate the downstream processes of osteoblast maturation [15–19]; they also interact and coordinately regulate the expression of bone-specific genes [45]. Moreover, BSP and OPN are two non-collagenous proteins which serve as a matrix-associated signals presenting overlapping and multifunctional activities in bone with effects on bone formation, mineralization and remodeling [44]. Although these BET induced signals might potentially result in increased production of a mineralized matrix, their ultimate outcomes need to be experimentally assessed in vivo given their known interaction with local and systemic regulatory factors.

This preliminary analysis of BET effect in bone cells has demonstrated the involvement of ERKs activation. ERK pathway represents an important signaling involved in osteogenesis [46, 47]. In bone, ERKs promote the proliferation and differentiation of osteoblasts [43, 48], and mediates the response of bone to a variety of stimuli, including hormone/growth factor stimulation [49, 50], extracellular matrix–integrin binding [51] and mechanical loading [52]. In particular, ERK phosphorylates RUNX2, thus enhancing RUNX2 dependent gene expression [15–19, 46, 47, 53].

As BET was able to depolarize the osteoblast membrane and to favour calcium influx by VDCCL, it is likely that BET could activate ERK via calcium signaling. In fact, it is generally acknowledged that calcium could activate ERK signaling via CaMKs [39, 54, 55]. Accordingly, we observed CaMKII signaling activation in hObs treated with BET (Fig. 5). Examples of exogenous molecules able to activate a calcium influx in hOBs by the L-type calcium channels are already known. Melittin for examples, present in the honeybee, [56] and casein phosphopeptides, originated from the casein digestion, [30] behave very similar to BET. Also 1,25(OH)_2_ Vitamin D3 acts on L-type calcium channels to activate the Ca^2+^ current requested to bone formation [42]. The possibility that food associated molecules could interact with L-type calcium channels and modulate the uptake of calcium by bone cells, can be considered as a potential tools in order to reach or maintain bone health.

Our study has shown that BET induces IGF-I production in bone cells, in agreement with previous observations indicating that IGF-I is a target of BET action. Apicella et al. have in fact reported that BET ingestion for 2 weeks resulted in an increase in plasma GH and IGF-I [5]. Moreover, BET was shown to directly enhance hepatocyte IGF-I secretion activating ERK pathway [57]. IGF-I is crucial for the development and maintenance of muscle and bone mass [58] and for the anabolic bone actions of growth hormone and parathyroid hormone [9]. The stimulation of IGF-I by BET in both osteoblasts and myoblasts suggest a potential modulatory effect of BET on bone-muscle cross-talk.

Changes in IGF-I pathway, cell calcium homeostasis and ERK signaling are tightly linked with enhanced oxidative stress and in particular with mitochondrial dysfunction [59]. Following the above consideration, our preliminary investigation showed that BET improves SOD2 gene expression and protein content, thus suggesting a possible role of BET in favouring bone response to bone cell stress condition. Further studies will be required to determine specific molecular pathways mediating BET effect on SOD2. This aspect is crucial in the hypothesis to use BET as nutraceutical supplement in patients with obesity or diabetes associated with osteoporosis and sarcopenia.

It is worth noting that primary culture of human osteoblast like cells is the first-line approach generally applied for the recognition of the potential effects of a particular substance on bone, as these cells are responsive to physiological and pharmacological stimuli and express several characteristics of human osteoblasts in vivo. Certainly, our in vitro findings will need further in vivo validation studies to better characterize the impact of BET on the benefit of people affected by bone and muscle mass loss.

## Conclusions

**Fig. 7:**
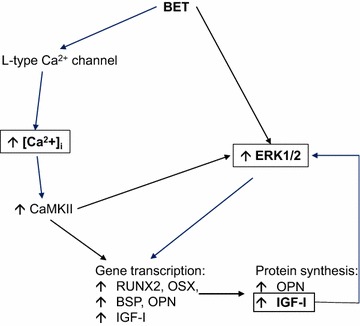
Schematic diagram of Betaine effect on human osteoblast

The present study has demonstrated that BET exerts a stimulatory effect on human osteoblast by acting on synergic pathways leading to osteogenic gene activation and production of bone matrix proteins (Fig. 7). This study has a translational value in opening the perspective that BET supplementation, by acting on bone and muscle cells through common pathways, might be effective in counteracting bone and muscle deterioration in the elderly, particularly in those individuals with an age-related pro-inflammatory state (i.e. obese and diabetic subjects). BET could represent an important nutraceutical approach in preventing the loss of muscle and bone with disuse, aging and disease, and in supporting therapies for age-related sarcopenia and osteoporosis [60], the major determinants of senile frailty and related mortality [61].

## Abbreviations

ALP: alkaline phosphatase
BET: betaine
BGP: osteocalcin
BSP: bone sialoprotein
CaMKII: Ca^2+^/calmodulin-dependent protein kinase II
DiBAC_4_-(3): bis-(1,3-dibutylbarbituric acid) trimethine oxonol
ERK1/2: extracellular signal–regulated kinase ½
FC: fold change
FBS: fetal bovine serum
Fura-2/AM: fura-2 acetoxymethyl ester
GAPDH: glyceraldehyde-3-phosphate dehydrogenase
hObs: human osteoblasts
IGF-I: insulin like growth factor I
mTOR: the mammalian target of rapamycin
OPN: osteopontin
OSX: osterix
p70 S6K: p70 S6 kinase
pCaMKII α: Ca^2+^/calmodulin-dependent protein kinase II isoform α
RUNX2: runt-related transcription factor 2
SOD2: superoxide dismutase 2
VDCCL: L-type calcium channels

## References

[1] Craig SA (2004). Betaine in human nutrition. Am J Clin Nutr.

[2] Lever M, Slow S (2010). The clinical significance of betaine, an osmolyte with a key role in, methyl group metabolism. Clin Biochem.

[3] Day CR, Kempson SA (2016). Betaine chemistry, roles, and potential use in liver disease. Biochim Biophys Acta.

[4] Cholewa JM, Guimarães-Ferreira L, Zanchi NE (2014). Effects of betaine on performance and body composition: a review of recent findings and potential mechanisms. Amino Acids.

[5] Apicella JM, Lee EC, Bailey BL, Saenz C, Anderson JM, Craig SA, Kraemer WJ, Volek JS, Maresh CM (2013). Betaine supplementation enhances anabolic endocrine and Akt signaling in response to acute bouts of exercise. Eur J Appl Physiol.

[6] Senesi P, Luzi L, Montesano A, Mazzocchi N, Terruzzi I (2013). Betaine supplement enhances skeletal muscle differentiation in murine myoblasts via IGF-1 signaling activation. J Transl Med.

[7] Burattini S, Ferri P, Battistelli M, Curci R, Luchetti F, Falcieri E (2004). C2C12 murine myoblasts as a model of skeletal muscle development: morpho-functional characterization. Eur J Histochem.

[8] Long F (2011). Building strong bones: molecular regulation of the osteoblast lineage. Nat Rev Mol Cell Biol.

[9] Govoni KE (2012). Insulin-like growth factor-I molecular pathways in osteoblasts: potential targets for pharmacological manipulation. Curr Mol Pharmacol.

[10] Xian L, Wu X, Pang L, Lou M, Rosen CJ, Qiu T, Crane J, Frassica F, Zhang L, Rodriguez JP, Xiaofeng J, Shoshana Y, Shouhong X, Argiris E, Mei W, Xu C (2012). Matrix IGF-1 maintains bone mass by activation of mTOR in mesenchymal stem cells. Nat Med.

[11] Fujita T, Azuma Y, Fukuyama R, Hattori Y, Yoshida C, Koida M, Ogita K, Komori T (2004). Runx2 induces osteoblast and chondrocyte differentiation and enhances their migration by coupling with PI3K-Akt signaling. J Cell Biol.

[12] Girgis CM, Baldock PA, Downes M, Vitamin D (2015). muscle and bone: integrating effects in development, aging and injury. Mol Cell Endocrinol.

[13] Kaji H (2014). Interaction between muscle and bone. J Bone Metab.

[14] Endo T (2015). Molecular mechanisms of skeletal muscle development, regeneration, and osteogenic conversion. Bone.

[15] Komori T (2006). Regulation of osteoblast differentiation by transcription factors. J Cell Biochem.

[16] Vimalraj S, Arumugam B, Miranda PJ, Selvamurugan N (2015). Runx2: structure, function, and phosphorylation in osteoblast differentiation. Int J Biol Macromol.

[17] Nishio Y, Dong Y, Paris M, O’Keefe RJ, Schwarz EM, Drissi H (2006). Runx2-mediated regulation of the zinc finger Osterix/Sp7 gene. Gene.

[18] Sinha KM, Zhou X (2013). Genetic and molecular control of osterix in skeletal formation. J Cell Biochem.

[19] Yang Y, Huang Y, Zhang L, Zhang C (2016). Transcriptional regulation of bone sialoprotein gene expression by Osx. Biochem Biophys Res Commun.

[20] Lai CF, Chaudhary L, Fausto A, Halstead LR, Ory DS, Avioli LV, Cheng SL (2001). Erk is essential for growth, differentiation, integrin expression, and cell function in human osteoblastic cells. J Biol Chem.

[21] Jaiswal RK, Jaiswal N, Bruder SP, Mbalaviele G, Marshak DR, Pittenger MF (2000). Adult human mesenchymal stem cell differentiation to the osteogenic or adipogenic lineage is regulated by mitogen-activated protein kinase. J Biol Chem.

[22] Bonjour JP (2011). Calcium and phosphate: a duet of ions playing for bone health. J Am Coll Nutr.

[23] Oliva J, Bardag-Gorce F, Tillman B, French SW (2011). Protective effect of quercetin, EGCG, catechin and betaine against oxidative stress induced by ethanol in vitro. Exp Mol Pathol.

[24] Sheng Y, Abreu IA, Cabelli DE, Maroney MJ, Miller AF, Teixeira M, Valentine JS (2014). Superoxide dismutases and superoxide reductases. Chem Rev.

[25] Görlach A, Bertram K, Hudecova S, Krizanova O (2015). Calcium and ROS: a mutual interplay. Redox Biol.

[26] Curtis E, Litwic A, Cooper C, Dennison E (2015). Determinants of muscle and bone aging. J Cell Physiol.

[27] Robey GP, Termine JD (1985). Human bone cells in vitro. Calcif Tissue Int.

[28] Montesano A, Luzi L, Senesi P, Mazzocchi N, Terruzzi I (2013). Resveratrol promotes myogenesis and hypertrophy in murine myoblasts. J Transl Med.

[29] Terruzzi I, Montesano A, Senesi P, Vacante F, Benedini S, Luzi L (2016). Ranolazine promotes muscle differentiation and reduces oxidative stress in C2C12 skeletal muscle cells. Endocrine.

[30] Donida BM, Mrak E, Gravaghi C, Villa I, Cosentino S, Zacchi E, Perego S, Rubinacci A, Fiorilli A, Tettamanti G, Ferraretto A (2009). Casein phosphopeptides promote calcium uptake and modulate the differentiation pathway in human primary osteoblast-like cells. Peptides.

[31] Grynkiewcz G, Poenie M, Tsien RY (1985). A new generation of Ca^2+^ indicators with greatly improved fluorescence properties. J Biol Chem.

[32] Fox J, Green DT (1986). Direct effects of calcium channel blockers on duodenal calcium transport in vivo. Eur J Pharmacol.

[33] Tsunoda Y, Stuenkel El, Williams AL (1990). Characterization of sustained [Ca^2+^]I increase in pancreatic acinar cells and its relation to amylase secretion. Am J Physiol.

[34] Pan CC, Cheng HH, Huang CJ, Lu YC, Chen IS, Liu SI, Hsu SS, Chang HT, Huang JK, Chen JS, Jan CR (2007). The antidepressant mirtazapine-induced cytosolic Ca^2+^ elevation and cytotoxicity in human osteosarcoma cells. Chin J Physiol.

[35] Merritt JE, Jacob R, Hallam TJ (1989). Use of manganese to discriminate between calcium influx and mobilization from internal stores in stimulated human neutrophils. J Biol Chem.

[36] Plasek J, Sigler K (1996). Slow fluorescent indicators of membrane potential: a survey of different approaches to probe response analysis. J Photochem Photobiol B.

[37] Wolff C, Fuks B, Chatelain P (2003). Comparative study of membrane potential-sensitive fluorescent probes and their use in ion channel screening assays. J Biomol Screen.

[38] Clapham DE (2007). Calcium signaling. Cell.

[39] Zayzafoon M (2006). Calcium/calmodulin signaling controls osteoblast growth and differentiation. J Cell Biochem.

[40] Zahanich I, Graf EM, Heubach JF, Hempel U, Boxberger S, Ravens U (2005). Molecular and functional expression of voltage-operated calcium channels during osteogenic differentiation of human mesenchymal stem cells. J Bone Mineral Res.

[41] Wen L, Wang Y, Wang H, Kong L, Zhang L, Chen X, Ding Y (2012). L-type calcium channels play a crucial role in the proliferation and osteogenic differentiation of bone marrow mesenchymal stem cells. Biochem Biophys Res Commun.

[42] Zanello LP, Norman A (2006). 1α,25 (OH)2 vitamin D3 actions on ion channels in osteoblasts. Steroids.

[43] Graham CS, Tashjian AH (1992). Mechanisms of activation of Na+/H+ exchange in human osteoblast-like SaOS-2 cells. Biochem J.

[44] McKee MD, Nanci A (1996). Osteopontin: an interfacial extracellular matrix protein in mineralized tissues. Connect Tissue Res.

[45] Pérez-Campo FM, Santurtún A, García-Ibarbia C, Pascual MA, Valero C, Garcés C, Sañudo C, Zarrabeitia MT, Riancho JA (2016). Osterix and RUNX2 are transcriptional regulators of sclerostin in human bone. Calcif Tissue Int.

[46] Ge C, Xiao G, Jiang DI, Franceschi R (2007). Critical role of the extracellular signal–regulated kinase–MAPK pathway in osteoblast differentiation and skeletal development. J Cell Biol.

[47] Greenblatt MB, Shim JH, Glimcher LH (2013). Mitogen-activated protein kinase pathways in osteoblasts. Annu Rev Cell Dev Biol.

[48] Franceschi RT, Ge C, Xiao G, Roca H, Jiang DI (2007). Transcriptional regulation of osteoblasts. Ann NY Acad Sci.

[49] Hurley MM, Marcello K, Abreu C, Kessler M (1996). Signal transduction by basic fibroblast growth factor in rat osteoblastic Py1a cells. J Bone Miner Res.

[50] Xiao G, Cui Y, Ducy P, Karsenty G, Franceschi RT (1997). Ascorbic acid-dependent activation of the osteocalcin promoter in MC3T3-E1 preosteoblasts: requirement for collagen matrix synthesis and the presence of an intact OSE2 sequence. Mol Endocrinol.

[51] Takeuchi Y, Suzawa M, Kikuchi T, Nishida E, Fujita T, Matsumoto T (1997). Differentiation and transforming growth factor-beta receptor down-regulation by collagen-alpha2beta1 integrin interaction is mediated by focal adhesion kinase and its downstream signals in murine osteoblastic cells. J Biol Chem.

[52] You J, Reilly GC, Zhen X, Yellowley CE, Chen Q, Donahue HJ, Jacobs CR (2001). Osteopontin gene regulation by oscillatory fluid flow via intracellular calcium mobilization and activation of mitogen-activated protein kinase in MC3T3-E1 osteoblasts. J Biol Chem.

[53] Qiao X, Nie Y, Ma Y, Chen Y, Cheng R, Yin W, Hu Y, Xu W, Xu L (2016). Irisin promotes osteoblast proliferation and differentiation via activating the MAP kinase signaling pathways. Sci Rep.

[54] Chao TS, Byron KL, Lee KM, Villereal M, Rosner MR (1992). Activation of MAP kinases by calcium-dependent and calcium-independent pathways. Stimulation by thapsigargin and epidermal growth factor. J Biol Chem.

[55] Hutchinson TE, Zhong W, Chebolu S, Wilson SM, Darmani NA (2015). L-type calcium channels contribute to 5-HT3-receptor-evoked CaMKIIα and ERK activation and induction of emesis in the least shrew (*Cryptotis parva*). Eur J Pharmacol.

[56] Chu ST, Cheng HH, Huang CJ, Chang HC, Chi CC, Su HH, Hsu SS, Wang JL, Chen IS, Liu SI, Lu YC, Huang JK, Ho CM, Jan CR (2007). Phospholipase A2-independent Ca2+ entry and subsequent apoptosis induced by melittin in human MG63 osteosarcoma cells. Life Sci.

[57] Lee MS, Kim MS, Park SY, Kang CW (2006). Effects of betaine on ethanol-stimulated secretion of IGF-I and IGFBP-1 in rat primary hepatocytes: involvement of p42/44 MAPK activation. World J Gastroenterol.

[58] Bikle DD, Tahimic C, Chang W, Wang Y, Philippou A, Barton ER (2015). Role of IGF-I signaling in muscle bone interactions. Bone.

[59] Cherry C, Thompson B, Saptarshi N, Wu J, Hoh J (2016). A ‘mitochondria’ odyssey. Trends Mol Med.

[60] Maggio M, De Vita F, Lauretani F, Buttò V, Bondi G, Cattabiani C, Nouvenne A, Meschi T, Dall’Aglio E, Ceda GP (2013). IGF-1, the cross road of the nutritional, inflammatory and hormonal pathways to frailty. Nutrients.

[61] Edwards MH, Dennison EM, Aihie Sayer A, Fielding R, Cooper C (2015). Osteoporosis and sarcopenia in older age. Bone.

